# Direct oral anticoagulant plasma concentrations according to the time elapsed since the last dose and to renal function: a retrospective study in geriatric patients with hip fractures

**DOI:** 10.1097/EA9.0000000000000098

**Published:** 2026-02-17

**Authors:** Erwan Milan, Kevin Serey, Dominique Fletcher, Charles Marc Samama, Augustin Schaefer

**Affiliations:** From the Sorbonne Université, Faculté de Santé, Paris, France (EM), Service d’Anesthésie – Réanimation Médecine Péri Opératoire, Hôpital Ambroise Paré (AP-HP, Boulogne-Billancourt) Université Paris Saclay, France (EM, KS, DF, AS), Service d’Anesthésie-Réanimation et de Médecine Péri-Opératoire, Hôpital Cochin, GHU APHP-Centre, Université Paris Cité, Paris, France (CMS)

## Abstract

**BACKGROUND:**

Early surgery for hip fractures in elderly patients is recommended to reduce morbidity and mortality. In patients treated with direct oral anticoagulants (DOACs), early surgical management raises pharmacological and safety concerns. Current guidelines recommend using plasma anticoagulant levels to guide perioperative management, but data describing the time course of DOAC plasma concentrations after the last dose in geriatric patients with hip fracture remain limited.

**OBJECTIVE(S):**

To describe the time course of DOAC plasma concentrations according to the estimated time since last intake in elderly patients admitted for hip fracture, and to compare residual concentrations at 24 h between patients with preserved and impaired renal function.

**DESIGN:**

Single-centre retrospective observational study.

**SETTING:**

Secondary care hospital; emergency admissions for hip fracture surgery. Study period: January 1st, 2022 to March 31st, 2025.

**PATIENTS:**

Patients aged ≥65 years treated with DOACs and admitted for hip fracture following a low-energy traumatic event. Patients receiving inappropriate DOAC dosing or DOACs despite formal contraindications were excluded from the main analysis.

**INTERVENTION(S):**

None.

**MAIN OUTCOME MEASURES:**

The primary outcome was the proportion of patients with a plasma DOAC concentration ≥100 ng ml^−1^ at 24 h after the last estimated dose. Renal impairment was defined as estimated glomerular filtration rate (eGFR) <50 ml min^−1^.

**RESULTS:**

Data from 186 patients were analysed. Among patients with eGFR ≥50 ml min^−1^, 1 out of 49 (2.0%) had a plasma DOAC concentration ≥100 ng ml^−1^ at 24 h. In contrast, 11 patients (9.4%) with eGFR <50 ml min^−1^ had concentrations ≥100 ng ml^−1^ at 24 h. Inappropriate DOAC dosing was significantly associated with higher residual plasma concentrations (9 patients excluded).

**CONCLUSIONS:**

Most elderly patients with preserved renal function had plasma DOAC concentrations below 100 ng ml^−1^ at 24 h. Although not powered to demonstrate definitive safety, these findings suggest that systematic plasma DOAC monitoring may be unnecessary in selected geriatric patients with hip fracture and preserved renal function. These results should be considered hypothesis-generating.

**TRIAL REGISTRATION:**

Not applicable (retrospective observational study).

**KEY POINTS**

- Data describing the time course of direct oral anticoagulant (DOAC) plasma concentrations after the last dose in geriatric patients admitted for hip fracture has remained limited.
- In our study, elderly patients with hip fractures treated with DOACs and preserved renal function had only exceptionally residual plasma concentrations above 100 ng ml^−1^ 24 h after the last dose.
- Impaired renal function (eGFR < 50 ml min^−1^) and inappropriate DOAC dosing were the main factors associated with persistently elevated plasma concentrations.
- Our findings suggest that routine plasma DOAC testing at 24 h may not be systematically required in selected geriatric patients with preserved renal function and appropriate dosing.

## Introduction

International clinical guidelines recommend that hip fractures should be treated within 48 h, as such early surgery has been associated with a lower risk of mortality and postoperative complications.^1^ As a result, in patients treated with direct oral anticoagulants (DOAC), this 48 h time limit may be too short to ensure sufficient elimination of the antithrombotic drugs.^2^ Plasma dosing of DOACs is suggested, but there is no consensus on the concentration threshold beyond which surgery is considered unreasonable as it varies across studies, typically ranging from 30 to 80 ng ml^−1^.^3^

Two pharmacokinetic studies have described concentration profiles of DOACs based on the time of the last dose: using data from the PAUSE study (which measured DOAC levels before scheduled surgery in 2451 patients) Shaw *et al.*^4^ found no overdosing (concentration > 400 ng ml^−1^) after 24 h, with only 0.5% of patients having levels above 100 ng ml^−1^. Godier *et al.*^5^ observed only one case of overdosing (out of 422 patients) after 24 h, with 93% of patients having levels below 100 ng ml^−1^. These studies also identified key predictive factors for delayed elimination: age >75 years, low body weight (<70 kg), drug interactions, and especially renal insufficiency.

Together with several reassuring retrospective clinical studies regarding prognosis and the use of transfusions in patients undergoing surgery 24 to 48 h after discontinuation of DOACs,^6,7^ these observations have led some teams to propose foregoing assessing DOAC plasma concentrations on admission for patients without renal insufficiency – provided that at least 24 h have passed since the last dose.^8^ However, compared with the usual population of hip fracture surgery patients, these pharmacokinetic studies were conducted with relatively younger patients (70–75 years vs. 82 years),^9^ less comorbidity (15% renal insufficiency, compared with nearly double after 80 years),^10^ and nonemergency settings. In fact, very little data exists on the elimination kinetics of DOACs in this population, even though >10% patients admitted for hip fracture are treated by DOACs.^11^

Our objective was to construct a plasma concentration profile based on the time of the last dose specifically for elderly patients admitted as emergencies, depending on the estimated time since their last dose, and then to compare this pharmacokinetic profile based on the presence or absence of renal insufficiency. Our hypothesis was that most patients without renal impairment would have a DOAC plasma levels compatible with surgery around 24 h after the last dose.

## Material and methods

### Ethics

This study complied with the MR-004 methodology of the French Data Protection Authority (CNIL) and received a favourable opinion from the Ethics Committee on Research in Anesthesia and Intensive Care (CERAR, Paris, France) on 24/03/2025 (IRB 00010254-2025-032, President: Dr Valerie BILLARD). Patient non-opposition was systematically sought before their final inclusion in the study. In accordance with applicable data protection regulations, patients who objected to the use of their medical records in a second step were not included in the study.

### Patients and study design

**Fig. 1:**
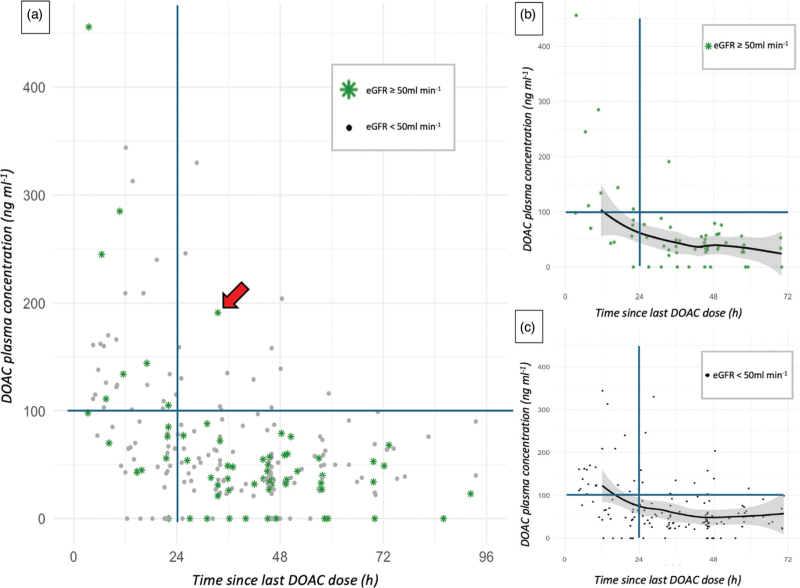
Plasma concentration profile of DOACs over time since the last dose (patients with inappropriate dosing excluded).

This was an internal study within the department of anaesthesiology of Ambroise Paré University Hospital (APHP, Boulogne-Billancourt, France). This single centre, retrospective trial was conducted solely with data available from the patient's electronic medical record (emergency admission reports, anaesthesia preoperative interviews, postoperative laboratory results). Eligible patients were identified from 1 January 2022 to 31 March 2025. The eligibility criteria were: any patient over 65 years of age, admitted for a femoral neck fracture after a low-energy mechanism of injury (e.g. a fall from standing), treated with direct oral anticoagulants (rivaroxaban, apixaban, or dabigatran), with available preoperative DOAC plasma levels (at least one dose). Exclusion criteria included discontinuation of DOAC therapy 72 h or more before hospital admission and a patient's refusal to allow the use of their data. In addition, the data for patients with contraindications for DOAC treatment, or noncompliance with dose adjustments for elderly patients recommended in France for DOACs^12^ were removed from the construction of concentration profiles (Fig. 1). These recommended dose adjustments were as follows: for apixaban, the dose was reduced to 5 mg per day in patients with an estimated glomerular filtration rate (eGFR) <30 ml min^−1^ or in the presence of two risk factors for overdose (age > 80 years, body weight < 60 kg, or serum creatinine > 1.5 mg dl^−1^); for rivaroxaban, the dose was reduced to 15 mg per day in patients with an eGFR < 45 ml min^−1^; for dabigatran, the dose was reduced to 220 mg per day in patients aged > 80 years. Indeed, in the absence of adjustment, some patients could receive twice the dosage as those of others (in the case of apixaban), which would introduce heterogeneity due to dosage regimens not aligned with current best practice recommendations.

Panel (a) overlays the concentration profile of patients with renal insufficiency (eGFR < 50 ml min^−1^, grey dots) and patients without renal insufficiency (green stars). In the latter group, only one patient had a concentration >100 ng ml^−1^ after 24 h (red arrow): this patient had Child A cirrhosis and had to manage his treatment alone despite cognitive impairment, but was retained in the analysis. Panels (b) and (c) separately show the concentration profile for patients with eGFR ≥50 ml min^−1^ and <50 ml min^−1^, respectively, with the trend line (and its confidence interval). Concentration profiles were constructed without patients with excessive DOAC dosing (nine patients). DOAC, direct oral anticoagulant; eGFR, estimated glomerular filtration rate.

The plasma DOAC levels routinely measured in our centre were interpreted according to various thresholds. A level of 400 ng ml^−1^ was considered as the threshold for “biological overdose” (beyond which spontaneous haemorrhages may occur).^13^ A level above 100 ng ml^−1^ was considered as a “high” level: there is very limited data regarding surgery in patients with high DOAC exposure. The “high” threshold was defined according to the work of Kaserer *et al.*,^14^ who retrospectively observed a significant increase in blood loss beyond this cut-off (and only in major procedures). That study, however, included only patients treated with rivaroxaban, and DOAC levels were estimated by an indirect pharmacokinetic model.

During time covered by the data collection, the protocol in place in our department required that surgery could only take place if a patient's plasma DOAC level was <50 ng ml^−1^ or at least 48 h had passed since the last DOAC dose. A concentration of 30 ng ml^−1^ was considered as the minimum threshold below which neuraxial anaesthesia was permitted in patients on DOACs.^15^

### Data collection

Eligible patients were identified from the hospital's surgical registries (hip prosthesis implantation, femoral neck fixation, or proximal nailing) and inclusion based on their anaesthesia records.

Clinical and biological data were collected: age, sex, weight, type of DOAC and its dosage, history of renal disease, history of liver cirrhosis, polypharmacy (defined as the use of five or more regular medications), time-to-surgery (time between hospital arrival and surgery), and any death within 1 month. The eGFR upon admission was estimated using the Cockcroft-Gault formula based on serum creatinine levels, as previously shown.^4,5^ Plasma DOAC concentrations were measured using commercially available, drug-specific assays routinely employed in our centre: a Biophen calibrated chromogenic anti-Xa assay (Hyphen Biomed, France) for rivaroxaban and apixaban, and the Hemoclot diluted thrombin time assay (Hyphen Biomed, France) for dabigatran.

Haemoglobin levels were recorded on admission and on day 1 postoperatively. The need for transfusion and the number of red blood cell units transfused were noted, along with the timing of the transfusion (pre, peri-, or postoperative). Since the exact time of the last anticoagulant dose is usually recorded by practitioners in the medical records as “morning” or “evening,” for the calculation of the interval between the last dose and plasma measurement, we considered that all morning doses were taken at 8 : 00 a.m., and evening doses at 8 : 00 p.m. for all patients.

### Statistical analysis

The primary objective of this study was descriptive (construction of a concentration profile in patients on DOAC) and did not require any calculation of the necessary sample size. However, we calibrated the study to compare the proportion of patients with a “high” DOAC level (>100 ng ml^−1^) persisting 24 h after the estimated time of the last dose, according to whether they had renal insufficiency upon admission (eGFR < 50 ml min^−1^) or not.

In the study by Godier *et al.*^5^ 7% of patients had levels above the 100 ng ml^−1^ threshold after 24 h. The odds ratio of having a concentration higher than the minimum threshold was approximately 3 for patients with renal insufficiency. Assuming an alpha risk of 0.05 and a beta risk of 0.8 and expecting similar variations (around 21% patients with concentration > 100 ng ml^−1^ for patients with renal insufficiency), we calculated that the required sample size was 94 patients per group to conduct statistical tests.

For the construction of curves, patients receiving excessive doses were excluded. The remaining patients could be represented multiple times on graphs (if their DOAC dose was measured multiple times). A nonparametric regression curve of the DOAC concentration values according to the estimated duration of DOAC discontinuation was built using the LOESS method, with a 95% confidence interval.

The prespecified secondary analyses were: comparison of the proportion of patients with a concentration >100 ng ml^−1^ after 24 h according to the estimated degree of renal insufficiency (eGFR < 25 ml min^−1^, eGFR 25 to < 50 ml min^−1^, eGFR ≥ 50 ml min^−1^) were followed. Transfusion rates and deaths within the first 30 days were reported for comparisons with external studies. Means were compared using the Wilcoxon test and proportions using Fisher's exact test. Quantitative variables are reported with their median (and interquartile range).

All tests were two-sided, with a *P* value of 0.05 considered significant. The computations were performed using R-Studio (version 6.2).

## Results

A total of 186 patients were included and their characteristics are summarised in Table 1.

**Table 1:** Patient characteristics and comparison between patients with a plasma concentration ≤100 ng ml^−1^ 24 h after last dose (low-levels) and those with a plasma concentration >100 ng ml^−1^ 24 h after last dose (high levels)

	All patients *n* = 186	Low-levels group DOAC concentration: ≤100 ng ml^−1^ 24 h after last dose *n* = 160	High-levels group DOAC concentration: >100 ng ml^−1^ 24 h after last dose *n* = 16	*P* value (univariate)
Age (years)	90 [87 to 95]	90.5 [87 to 98]	89.5 [86 to 94]	0.48
Female sex (%)	138 (74.2)	117 (73)	11 (68)	0.77
Weight (kg)	60 [50 to 71]	61 [47 to 71]	56.5 [49 to 65.5]	0.78
eGFR* (ml min^−1^)	39.5 [26 to 51.6]	**40.2** [27.7 to 52.8]	**28** [21.7 to 43.3]	**0.039**
- eGFR > 50 (%)	51 (27.4)	49 (30.6)	2 (12.5)	
- eGFR 50–25 (%)	88 (47.3)	80 (50)	8 (50)	
- eGFR < 25 (%)	27 (14.5)	21 (13.1)	6 (37.5)	
Cirrhosis (%)	2 (1.1)	0 (0)	2 (12.5)	N.A
Prolonged lying on the ground (%)	7 (4)	7 (4.5)	0 (0)	N.A
Polypharmacy (%)	150 (80)	132 (82)	14 (87.5)	1
Anticoagulant taken (%)				
- Apixaban	148 (79.6)	123 (76)	16 (100)	
- Rivaroxaban	35 (18.9)	34 (21.2)	0 (0)	
- Dabigatran	3 (1.6)	3 (1.8)	0 (0)	
Excessive prescribed dose (%)	9 (4.8)	**5 (3.1)**	**4 (25)**	**0.006**
Preoperative haemoglobin (g dl^−1^)	12.3 [7.1 to 12.7]	12.3 [7.1 to 12.4]	12.2 [6.8 to 12.4]	0.66
Surgery (%)				
- Gamma nail	110 (59.1)	98 (61.2)	7 (43.7)	
- IHR	59 (31.7)	50 (31.2)	8 (50)	
- THR	11 (5.9)	9 (5.6)	1 (6.2)	
- Hip screw	2 (1.1)	2 (1.3)	0 (0%)	
Time to surgery (h)	58.6 [47.7 to 77.8]	**58.6** [47.7 to 81.9]	**78.5** [49.2 to 84.2]	**0.006**
Transfusion rate (%)	79 (42.4)	60 (37.5)	12 (75)	
- Before surgery	15 (18)	12 (20)	3 (18.7)	
- During surgery	35 (44)	36 (60)	7 (43.7)	
- After surgery	46 (58)	24 (40)	8 (50)	
Total number of units transfused	1 [0–2]	**0** [0–2]	**2** [0–3]	**0.004**
Mortality at day 30 (%)	9 (4.4)	8 (5)	1 (5.9)	0.58

Data are presented as median [IQR] or number (%). “Polypharmacy” is defined as the use of five or more regular medications, “prolonged lying on the ground” is defined as a patient having remained on the ground for >12 h without being able to get up before help arrived. An “excessive prescribed dose” was defined as a daily DOAC regimen that did not comply with the dose adjustments recommended in France for elderly patients.^12^ “Time to surgery” was defined as the interval between admission to the Emergency Department and surgical incision.

The last column shows the *P* value for the comparison between the high and low levels groups, performed using the Wilcoxon or Fisher tests according to the nature of the variable. Bolded values indicate a statistically significant difference.

Note: 10 patients included in the study could not be classified into either the High level or low level group (due to an initial concentration >100 ng ml^−1^ <24 h after the last dose and no subsequent measurement after 24 h).

DOAC, direct oral anticoagulant; eGFR, estimated glomerular filtration rate; IHR, intermediate hip replacement; THR, total hip replacement.

Ten patients included in the study could not be classified into either the “high level” or “low level” group (due to an initial concentration > 100 ng ml^−1^ less than 24 h after the last dose and no subsequent measurement after 24 h).

Due to inappropriate DOAC dosage regimens not being in accord with recommendations, 9 patients (4.8%) were excluded from the construction of concentration–time curves according to the time elapsed since the last dose. Reduced DOAC dosing, in accordance with guideline recommendations (advanced age, low body weight, or renal impairment), was found in 123 patients (66.1%).

The plasma concentration profile of DOACs over time since the last dose is shown in Fig. 1. After excluding patients with an excessive prescribed dose, only one patient (2.0%) with an eGFR >50 ml min^−1^ had a DOAC level >100 ng ml^−1^ 24 h after the last dose, compared with 11 (9.4%) for patients with impaired eGFR. The number of patients in the “high level” group was surprisingly small when compared with the study by Godier *et al.*,^5^ which served as the basis for our power calculation (21% expected in the presence of renal impairment). Despite a large relative difference, this difference did not reach statistical significance (*P* = 0.1).

The patients with inappropriate dosages were significantly overrepresented in the group with high plasma DOAC levels (25% of patients, compared with 3.1% in the low level group).

Only 2 patients had cirrhosis: one had decompensated cirrhosis (Child C11) and had to be excluded for receiving an inappropriate dosage. The second (Child A6) had cognitive disorders and social isolation. However, he was retained in the analysis. Both had persistent elevated levels after 24 h, but no conclusion can be drawn from such a small number of observations.

The use of transfusion during the hospital stay was high in our population (42% in total – with over 18% preoperatively) and occurred significantly more often in the high level group.

The median time between admission and surgery was 58 h, and it was significantly higher in the high level group. It was not possible to show a difference in the proportion of patients who died at 30 days.

## Discussion

This retrospective study enabled us to construct a drug concentration profile for almost all patient who had undergone surgery for femoral neck fractures and who were being treated with DOACs in our centre since 2022. It showed that in the presence of an eGFR >50 ml min^−1^ at admission, the likelihood of having a level >100 ng ml^−1^ is rare (1 patient out of 49, after excluding patients with excessive dosages) and may therefore be generalisable to other populations.

Compared with previous pharmacokinetic studies on DOACs,^4,5^ our work specifically adds to available evidence by focusing on a very elderly population (median age 90 years) admitted in an emergency setting, for which very little data were available.

Across our cohort, we were surprised by the proportion of patients with DOAC plasma levels >100 ng ml^−1^: based on the results of previous teams we would have expected two to three times more such patients, and even more, given that we studied an older population, with more comorbidities managed in an emergency setting.

One possible explanation for this unexpected finding is the high proportion of patients receiving adjusted dosage schedules (66.1%). It has previously been demonstrated that dose reduction in elderly patients does not compromise the efficacy of DOAC therapy.^16^ Our findings may also suggest that such dose adjustments are beneficial when urgent surgery becomes necessary, as circulating levels fall more rapidly into a range compatible with operative intervention.

These observations, although retrospective, were derived from patient medical records: the timing of the last dose was based on information provided by the patient or their relatives and documented by the emergency department team. While this inevitably introduces a measurement bias and limits precision compared with previous pharmacological studies, these are “real-world” observations, which are generally the only data available in clinical practice.

Similarly, this study relies on an estimate of eGFR based on serum creatinine levels at admission. However, eGFR estimates may be unreliable in the setting of acute kidney injury, a situation commonly encountered in geriatric patients in this context, particularly due to dehydration.^17^ In such cases, the measured eGFR at admission may overestimate the patient's true renal function at the time of surgery. Consequently, a “no-test” strategy could be associated with an increased risk in patients with borderline renal function (eGFR just above 50 ml min^−1^), who may experience delayed DOAC elimination if renal function deteriorates preoperatively. This concern is especially relevant in patients at higher risk of functional acute kidney injury, such as those with prolonged immobilisation or dehydration. However, this estimate is often the only available data for the practitioner managing the patient in this emergency context.

The main clinical implication of our study is that it supports the rationale for omitting routine DOAC plasma testing for some patients. Our results should enable discussion of strategies already proposed by some teams (with the limitation that our work does not provide justification for the choice of any particular concentration as a threshold for safe surgery). For example, the protocol at Leicester Hospitals^8^ suggests omitting testing for patients with an eGFR >30 ml min^−1^. In our study, we observed (after excluding overdosed patients) that nine patients (25% of those with eGFR > 30 ml min^−1^) had a level >100 ng ml^−1^ after 24 h – whereas the British protocol proposes this same threshold as the operability limit when the test is performed.

The idea of omitting plasma DOAC measurement to decide on operability is indeed not new: Aziz *et al.*^18^ showed that a strategy based on repeated measurements every 24 h until the desired threshold is reached doubled the average surgery delay and transfusion risk compared with patients operated on without testing, after 24 to 48 h of anticoagulant discontinuation. Therefore, performing a test may be best reserved for patients at high risk of overdose in the context of a femoral neck fracture – in our study, these were patients with impaired renal function. We would also add to this group those patients who are likely to receive inappropriate doses: excessive dosage regimens or self-management of treatment despite cognitive disorders.

Only two variables were significantly associated with the “high level” group: an eGFR <50 ml min^−1^ and excessive DOAC dosing relative to guideline recommendations. Interestingly, although no firm conclusion can be drawn, both patients who had liver disease (cirrhosis), had levels >100 ng ml^−1^ after 24 h. The patient included in the analysis had no renal function impairment but presented cognitive disorders and social isolation. Medication errors in this patient, who managed his own treatment despite cognitive disorders, cannot be excluded. This is consistent with the pharmacology of DOACs:^19^ apixaban and rivaroxaban undergo substantial hepatic metabolism (≈75% and 65% respectively, via CYP3A4/5 and CYP2J2), whereas dabigatran is primarily eliminated by the kidneys (~80%) with minimal hepatic involvement. Hepatic dysfunction may therefore disproportionately impair the elimination of apixaban and rivaroxaban. Although our data are limited, these observations suggest that systematic DOAC testing could be advisable with any degree of liver disease.

Several limitations must be acknowledged. First, the study was underpowered, since the rate of patients with residual levels >100 ng ml^−1^ was lower than expected (2% vs. 7% expected in the group without renal insufficiency, 9.4% vs. over 21% expected in the group with renal insufficiency). This may explain the lack of statistical significance in the difference. The number of patients with normal or only mildly impaired renal function (eGFR > 50 ml min^−1^) was also small: 49 (27.8%) of assessed patients. This finding was not unexpected given the advanced age of the study population (median age 90 years), even compared with typical patients treated for hip fracture. However, this may limit the possibility of omitting DOAC level monitoring in this population.

Second, this study was not designed to investigate the prognosis of patients (especially since all were treated according to the same protocol: surgery only took place if the DOAC level was < 50 ng ml^−1^ or after 48 h of anticoagulant discontinuation). Observations regarding mortality or transfusion criteria should therefore be cautious. It can be noted that the transfusion rate is high, though not surprising: several studies have found rates as high, up to 75%.^20^ A significant portion of transfusions were performed preoperatively (about 20%) in our study, probably in anticipation of surgery but possibly also due to bleeding from the fracture site before operation. The time to surgery, reported for descriptive purposes, was very long and well above general recommendations. Our surgical team has not implemented a “fast-track” pathway, and other groups using cautious protocols for patients on DOACs have reported similarly prolonged delays, much longer than those observed in patients receiving other types of antithrombotic therapy.^21^ Unsurprisingly, patients in the high level group had a significantly longer delay, but it was not possible to show an impact on 30-day mortality, which remained close to the perioperative mortality rates seen in other studies.^22^ If confirmed, this lack of increased mortality would also not be surprising: Brameier *et al.*^7^ showed that patients on DOACs operated on beyond the recommended time (after 48 h) did not have higher mortality. Performing surgery earlier, however, significantly reduced transfusion needs and length of hospital stay.

Finally, our study also included very few patients on dabigatran (only three, and none in the “high level” group): this point is potentially problematic, since Dabigatran is primarily eliminated via the kidneys (unlike apixaban or rivaroxaban which undergo mainly hepatic metabolism and elimination). Therefore, even mild impairments in renal function may significantly prolong the time required to reach a concentration below 100 ng ml^−1^.^23^ Caution is warranted when extrapolating our results to patients receiving this DOAC.

Studies comparing the prognosis of patients operated on at different DOAC concentration thresholds are rare. We based our choice of the 100 ng ml^−1^ threshold on the observations of Kaserer *et al.*^14^ This threshold is higher than that previously used in our institution (50 ng ml^−1^), which seemed too restrictive in light of the results of Hofer *et al.*^24^ This latter group had compared the prognosis of patients with hip fractures operated on urgently at various thresholds (30, 50 and 80 ng ml^−1^), without showing any differences except for a slight increase in transfusion needs for patients over 80 ng ml^−1^, which they attributed to a higher incidence of preoperative anaemia. Their work involved a patient population similar to ours, with 30% of their patients operated on with plasma levels >50 ng ml^−1^. Their findings are reassuring with respect to our hypothesis that surgery after 24 h can be performed safely in patients without renal impairment, since our data indicate that DOAC concentrations are unlikely to exceed 100 ng ml^−1^. However, no study has yet focused on the 100 ng ml^−1^ threshold specifically for patients admitted for hip fracture.

In conclusion, this study provides further insight into the DOAC concentration profile in patients hospitalised urgently for femoral neck fractures. Elimination appears quite predictable in patients without renal insufficiency (eGFR ≥ 50 ml min^−1^ at admission), suggesting that plasma DOAC testing may be unnecessary for selected patients if 24 h have passed since their last dose. However, practitioners wishing to adopt this strategy – which needs to be confirmed by interventional studies – should ensure that their patient has received the appropriate dosage and perform the test if there is any doubt about compliance.
